# Elevated IL-17 levels in semi-immune anaemic mice infected with *Plasmodium berghei* ANKA

**DOI:** 10.1186/s12936-018-2257-x

**Published:** 2018-04-17

**Authors:** Gideon Kofi Helegbe, Nguyen Tien Huy, Tetsuo Yanagi, Mohammed Nasir Shuaibu, Mihoko Kikuchi, Mahamoud Sama Cherif, Kenji Hirayama

**Affiliations:** 1 Department of Immunogenetics, Institute of Tropical Medicine (NEKKEN), Nagasaki University, 1-12-4 Sakamoto, Nagasaki, 852-8523 Japan; 2 Department of Biochemistry and Molecular Medicine, School of Medicine and Health Sciences, University for Development Studies, Tamale, Ghana; 3 West Africa Center for Cell Biology of Infectious Pathogens (WACCBIP), Department of Biochemistry, Cell and Molecular Biology, University of Ghana, Accra, Ghana; 4 National Bio-Resource Center (NBRC), NEKKEN, Nagasaki University, 1-12-4 Sakamoto, Nagasaki, 852-8523 Japan; 5 Department of Biochemistry, Ahmadu Bello University, Zaria, Nigeria

**Keywords:** IL-17, Semi-immune mice, Anaemia, *Plasmodium berghei* ANKA

## Abstract

**Background:**

Alterations in inflammatory cytokines and genetic background of the host contribute to the outcome of malaria infection. Despite the promising protective role of IL-17 in infections, little attention is given to further understand its importance in the pathogenesis of severe malaria anaemia in chronic/endemic situations. The objective of this study, therefore, was to evaluate IL-17 levels in anaemic condition and its association with host genetic factors.

**Methods:**

Two mice strains (Balb/c and CBA) were crossed to get the F1 progeny, and were (F1, Balb/c, CBA) taken through 6 cycles of *Plasmodium berghei* (ANKA strain) infection and chloroquine/pyrimethamine treatment to generate semi-immune status. Cytokine levels and kinetics of antibody production, CD4^+^CD25^+^T regulatory cells were evaluated by bead-based multiplex assay kit, ELISA and FACs, respectively.

**Results:**

High survival with high Hb loss at significantly low parasitaemia was observed in Balb/c and F1. Furthermore, IgG levels were two times higher in Balb/c, F1 than CBA. While CD4^+^CD25^+^ Treg cells were lower in CBA; IL-4, IFN-γ, IL-12α and IL-17 were significantly higher (p < 0.05) in Balb/c, F1.

**Conclusions:**

In conclusion, elevated IL-17 levels together with high IL-4, IL-12α and IFN-γ levels may be a marker of protection, and the mechanism may be controlled by host factor (s). Further studies of F2 between the F1 and Balb/c will be informative in evaluating if these genes are segregated or further apart.

**Electronic supplementary material:**

The online version of this article (10.1186/s12936-018-2257-x) contains supplementary material, which is available to authorized users.

## Background

Malaria, a protozoan disease caused by parasites of the genus *Plasmodium*, continues to be a major public health threat worldwide. There are currently six known species (*Plasmodium falciparum*, *Plasmodium vivax*, *Plasmodium malariae*, *Plasmodium knowlesi*, *Plasmodium ovale wallikeri* and *P. o. curtisi*) [1], that cause malaria in human. Of these *Plasmodium* species, *P. falciparum* is the leading cause of death in the tropics. According to the latest World Health Organization estimates, there were 212 million cases of malaria in 2015 and 429,000 deaths due to *P. falciparum* [2]. Acquiring immunity to malaria is dependent on age and repeated infections an individual has had. Therefore, in endemic areas, adults usually show a symptomatic form of the disease and in some instances, are chronically infected with low parasitaemias [3]. In areas with high transmission of malaria, children under 5 are particularly susceptible to infection, illness and death; more than two-thirds (70%) of all malaria deaths occur in this age group. Most of the deaths are due to complications from the severe forms of malaria, i.e. severe malaria anaemia (SMA), cerebral malaria and intra-vascular haemolysis (IVH) [4]. Between 2010 and 2015, the under-5 malaria death rate fell by 29% globally. However, malaria remains a major killer of children under 5 years old, taking the life of a child every 2 min.

Several studies have reported on the role played by anti- and pro-inflammatory cytokines in the pathogenesis of malaria [5–11]. Furthermore, high levels of some pro-inflammatory cytokines (e.g., IFN-γ and TNF) have been observed to be protective [10, 12]. Though little attention has been given to IL-17 in malaria infection few studies have shown that IL-17 is needed for IL-23 to offer protection against *Plasmodium berghei* (NK65 strain) infection [13]; significant expansion of IL-17 producing cells correlated to a pro-inflammatory cytokine profile in *Plasmodium vivax* infection [14]; high IL-17 is associated with high mortality in *P. berghei* (ANKA strain) infections [15]. In addition to its role in host defence against extracellular bacterial infection, IL-17 has been shown to be important in protection against fungal and parasitic infection. IL-17R-deficient mice were reported to have increased kidney fungal burden and decreased survival upon *Candida albicans* challenge [16]. The paradox about IL-17 is that it is both protective and pathological. The balance between protection and pathologic consequences was seen in the association between *Helicobacter pylori*, IL-17 and damage to gastric mucosa that leads to ulceration [17]. IL-17 has been shown to be involved in central nervous system (CNS) diseases [18]. In that study, the evidence supplied indicates that inhibiting the function of the IL-17 cytokine family could have a beneficial effect on pathogenic conditions in the CNS.

Anaemia in malaria has been extensively studied and documented. Mechanisms proposed to be involved in the anaemia in malaria have ranged from rupture of infected red blood cell (iRBC) due to parasite proliferation [19], immune mediated dependent destruction of RBC (antibody, complement, cytokines, cellular) [20–24] to suppression of the bone marrow [25]. A study has shown variation of IL-17 levels in different semi-immune mice strains implicating a role of IL-17 in RBC loss due to *Plasmodium* infection [24]. Recent reports have also shown IL-17 relationship with SMA [26, 27]. Apart from these studies linking IL-17 with haemoglobin (Hb) loss, other studies have shown IL-17 to be involved in multiple organ dysfunction [28], and also found to be associated with higher risk in developing cerebral malaria (CM) [29]. Since previous studies have reported the recovery of a group of semi-immune mice at low parasitaemia [23, 24], and also for the fact that IL-17 was implicated in one study [24], it is hypothesized that IL-17 is involved in the recovery/protection of the semi-immune mice at low Hb levels. The aim of this study, therefore, was to assess levels of IL-17 in anaemic condition as well as evaluate its association with host genetic factors. A significance for this current study is that change in levels of IL-17 and its association with other immunological parameters can be exploited as candidates for disease biomarkers and possible therapy in malaria anaemia.

## Methods

### Mice, infection and generation of semi-immune status

**Fig. 1:**
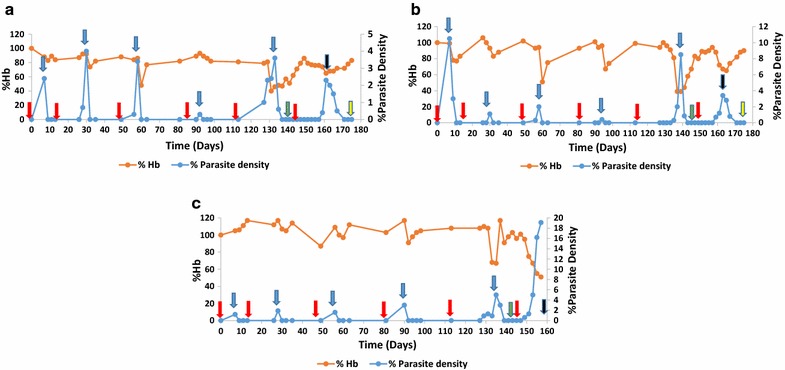
Profile of parasitaemia and haemoglobin levels in the semi-immune mice strains during the five cycles of infection and treatment. Upper left graph **a** refers to profile for F1; upper right graph **b** refers to profile for Balb/c and lower graph **c** refers to profile of CBA. The data presented here is representative of one mouse per strain/group. Arrows in red implies infection with 10^4^ *Plasmodium berghei* (ANKA strain); arrows in blue signifies treatment schedules with chloroquine/pyrimethamine; arrows in green refers to blood harvested or mice sacrificed (for T cell assessment) on D0; black arrows implies harvesting of blood or sacrificing of mice (for T cell assessment) at infection (D12 for CBA, D16 for F1 and Balb/c); yellow arrow refers to blood harvested or mice sacrificed (for T cell assessment) during recovery (i.e. parasite density is zero by microscopy), D28 for F1 and Balb/c

Previous studies [21, 22, 30] indicated destruction of relatively high uninfected red blood cells (uRBC) in Balb/c semi-immune mice at low parasite density, while relatively low uRBC were destroyed in CBA. Uninfected RBC destruction was implicated as no parasite sequestration was observed in any organs (spleen, liver, brain, kidney, lung, heart and muscle) [30]. Based on this observation Balb/c (described as the more destructive) and CBA (less destructive type) were chosen for the study. And to understand if any gene is involved in this phenomenon, the F1 cross between Balb/c and CBA was made. The procedure for this method has been reported elsewhere [23, 24]. Briefly, 8-week-old female Balb/c, CBA and F1 (Balb/c × CBA) mice supplied by SLC laboratories, Fukuoka, Japan, were injected intraperitoneally (i.p.) with 10^4^ *P. berghei* (ANKA strain) infected red blood cells. This line of *P. berghei* ANKA was generously donated by Dr. Tetsuo Yanagi, of National Bio-Resource Center (NBRC), NEKKEN, Nagasaki University, Nagasaki, Japan. For every 2 days, levels of parasitaemia and reticulocyte were monitored by Giemsa-stained thin blood film. These were expressed as a percentage of more than 500 RBCs. Even though a recent study [31] has shown that estimates of polychromatophilic cells as done after Giemsa staining in this study are typically less than percentages of reticulocytes, Giemsa has been successfully used previously for reticulocyte staining [23, 24, 32]. Method used in estimating the level of blood haemoglobin (Hb) is as previously described [23]. By day 6 after infection, chloroquine (10 mg/kg i.p.) and pyrimethamine (10 mg/kg i.p.) were used to treat mice daily for 6 days. This infection and treatment of the mice were done for 5 cycles. This was to ensure mice attain semi-immune status. A mouse is considered semi-immune due to the several cycles of infection and treatment. An additional parameter is the observation of lower parasitaemia growth during other cycles of infection in relation to the first cycle (Fig. 1). Between each round of infection (after the first cycle), mice were rested for 2 weeks before being re-challenged with 10^4^ *P. berghei*. The mice were then monitored and drug-cured before parasitaemia attains 10%. A graphic outline of the infections, chemotherapy and time point of analysis are indicated in Fig. 1. Parasitaemia and haemoglobin profile in the semi-immune mice strains was similar to that described previously [24]. For baseline value, blood was collected from the tail after the 5th cycle of infection and treatment, after parasitaemia was checked to be negative (zero). Serum was harvested from the tail blood before the final cycle of infection without treatment. Mice were monitored every other day during the final cycle of infection (without treatment). Blood harvesting was done on days in which minimum Hb (i.e. maximum Hb reduction) was observed, while the procedure for serum preparation and storage is as described previously [23].

### Measurement of antibody titer using ELISA

Immunoglobulin G and its subtype antibody responses were assessed by ELISA from the sera collected after the fifth cycle. Briefly, 96-well plates were coated with 100 μL of 0.5 μg/mL of *P. berghei *crude antigen in coating buffer and kept overnight at 4 °C. Plates were washed three times with 400 μL/well of 0.05% Tween-PBS (phosphate-buffered saline) and then blocked for nonspecific binding using 340 μL/well of 0.1% blocking reagent (Roche Diagnostics, Mannheim, Germany) for 1 h at 37 °C. Plates were washed three times with 400 μL/well of 0.05% Tween-PBS and 100 μL of serially diluted pooled sera (1:80) was added and incubated at 37 °C for 3 h. Plates were then washed five times with 400 μL/well of 0.05% Tween-PBS, and 100 μL of horseradish peroxidase (HRP)-conjugated goat anti-mouse IgG and subclasses IgG1, IgG2a and IgG3 (Southern Biotechnology, Birmingham, AL) diluted with blocking buffer (1:2500) was added and incubated for 1 h at room temperature. Plates were washed 5 times with 400 μL/well of 0.05% Tween-PBS and antigen–antibody reaction was visualized by the addition of 50 μL/well of 3, 3′, 5, 5′-tetramethylbenzidine (TMB) (Vector Laboratories, CA, USA). The colour development reaction was stopped after 30 min by adding 50 μL of 1 N of H_2_SO_4_, and the absorbance was measured in an automated plate reader (Bio-Rad, Hercules, CA) at 450 nm.

### Analysis of splenocytes populations by flow cytometry (FACs analysis)

Spleens were excised from individual mouse during the sixth cycle of infection without treatment (days zero for the mice strains used in the experiment; days 12 for CBA; days 16 and 28 for F1 and Balb/c). The frequency of CD4(+) CD25(+) Treg cell was measured by FACS. Analysis of cell surface markers (CD3, CD4, and CD25) from BD Pharmingen (BD Biosciences San Jose, California, USA), intracellular cytokine (Foxp3) expression recognition were done. Fluorescent-conjugated monoclonal antibodies, and isotype control were purchased from Biolegend (San Diego, California, USA). Purified Rat Anti Mouse CD16/CD32 antibody was purchased from BD Biosciences Pharmingen (San Jose, California, USA) to block non-antigen-specific binding of immunoglobulins to the FcγIII and FcγII. Cells were stained with CD3, CD4, CD25 surface markers. Cells were then fixed for 20 min at 4 °C in 4% paraformaldehyde, washed and permeabilized in the presence of 0.1% saponin. Finally, re-suspended cells were incubated for 30 min at 4 °C in the presence of optimal concentrations of Foxp3 and conjugated rat IgG2a isotype controls were used for the intracellular staining. The Tregs frequency was evaluated by flow cytometry using Becton Coulter Gallios, Flow Cytometer (Beckman Coulter, Inc.), and data were analysed using Kaluza software.

### Inflammatory cytokines measurement

This method as described previously [23] was done according to the manufacturer’s instructions. Cytokines measurement was done using Procarta Mouse Cytokine Assay Kit plex according to manufacturer’s instructions (Luminex, Affymetrix). Briefly, after the reading buffer was used to wet the plate, it (the plate with the reading buffer) was incubated at room temperature for 5 min, and later filtered. To each well was added antibody beads (50 μL), then filtered and washed once with washing buffer (150 μL). This procedure was followed by the addition of 25 μL of serum standard buffer to all the sample wells, followed with the addition of equal volume (25 μL) of serum to each well, and then incubated for 60 min at room temperature. Washing was done three times, later followed with the addition of 25 μL premixed detection, and then incubated for 30 min on the shaker at room temperature. Washing and filtration was done three times afterwards. One hundred and fifty (150) μL of washing buffer was used during each wash. Washing and filtration was done three times after incubation was done for 30 min with Streptavidin-PE (50 μL).The plate was prepared for analysis after the third wash, with the addition of 120 μL reading buffer.

### Data analysis

GraphPad Prism Version5.00 for Windows, GraphPad Software, San Diego California, USA, [Graph pad prism version 5.0 for Windows; http://www.graphpad.com] was used for the data analysis. Data are expressed as the mean with standard deviation (SD) unless otherwise stated. To ensure normal distribution, data were log transformed before one-way analysis of variance (ANOVA, with Tukey’s post-test, two tailed), were performed. The differences of means were considered statistically significant if p < 0.05.

### Ethical statement

The Fundamental Guidelines for Proper Conduct of Animal Experiment and Related Activities in Academic Research Institutions under the jurisdiction of the Ministry of Education, Culture, Sports, Science and Technology, Japan (Notice no. 71) was strictly followed during the study. All efforts were made to humanely minimize animal suffering. The Nagasaki University, Board of Animal Research approved all animal experiments, according to Japanese Guideline for use of Experimental animals (Permit Number 0811130716).

## Results

### Mortality, parasitaemia-time course, profile of haemoglobin loss and erythropoietic response in the semi-immune ice strains

**Fig. 2:**
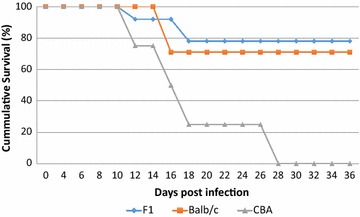
Cumulative survival curve for the semi-immune mice strains. Death of mice were monitored after challenge with 10^4^ *P. berghei* following attainment of semi-immune status in the semi-immune mice strains without treatment. F1, n = 10; Balb/c, n = 7; CBA, n = 4

**Fig. 3:**
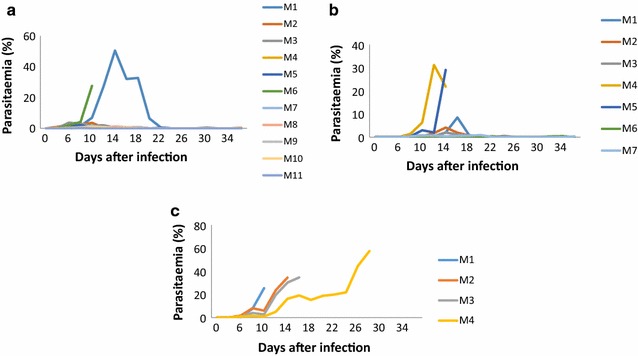
Trend of parasitaemia in the semi-immune mice. Values indicated are representative data of infected semi-immune mice: **a** F1, n = 10; **b** Balb/c, n = 7; **c** CBA, n = 4. This trend of parasitaemia is reflective of infection with 10^4^ *P. berghei* during the final cycle without treatment

Balb/c, CBA and the cross between them called F1 were taken through five cycles of infection with 10^4^ *P. berghei* followed by pyrimethamine and chloroquine treatment to generate the semi-immune status. After the fifth cycle, these semi-immune mice strains were challenged with 10^4^ *P. berghei* without treatment to observe how the semi-immune status of the mice can be measured. Figure 2 revealed that, while all the CBA mice were dead by day 28 (probably due to high parasitaemia, Fig. 3c), most of F1 and Balb/c mice survived, Fig. 2.

**Fig. 4:**
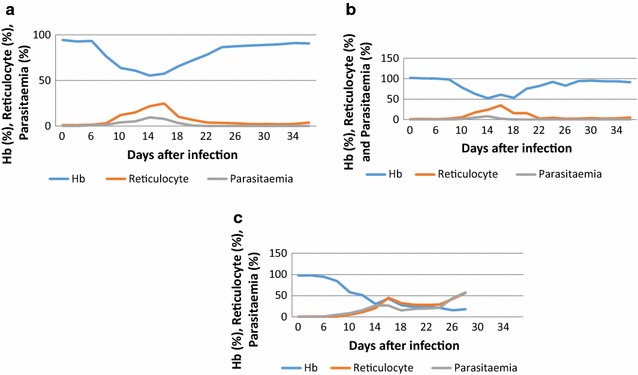
Malaria anaemia profile in the semi-immune mice strains. Values shown are mean of Hb, reticylocyte and parasitaemia in the semi-immune mice strains: **a** F1, n = 10; **b** Balb/c, n = 7; **c** CBA, n = 4, after *P. berghei* infection during the final cycle. Values as indicated are representative data of one experiment

Figure 3 shows the parasitaemia in the semi-immune mice strains in another experiment after the mice strains were challenged with 10^4^ *P. berghei*. It can be seen that prepatent period was 4 days in the mice strains (Fig. 3a–c). Another interesting observation made is that while high parasitaemia (50% for F1 and 30% for Balb/c) were also recorded by some mice in the F1 and Balb/c mice, some recovered and a few died (Fig. 3a [M6 mouse], b [M4 and M5 mice]). Much higher parasitaemia was, however, observed in the individual mice strains of CBA (Fig. 3c). It was also observed that the semi-immune mice strains of CBA died, even though some mice appeared to resolve their parasitaemia (Fig. 3c), they could not fully recover. The mean blood haemoglobin, reticulocytes, and parasitaemia kinetic profiles of F1, Balb/c and CBA strains were also recorded as shown in Fig. 4. A clear observation made was that an Hb decrease and an increase of reticulocyte count was associated with peak parasitaemia. Hb improved gradually within F1 and Balb/c semi-immune mice while parasites were cleared as well within these semi-immune mice strains (F1 and Balb/c) (Fig. 4a, b). On the other hand CBA could not clear the parasites resulting in continued fall in Hb and eventual death (Fig. 4c).

**Fig. 5:**
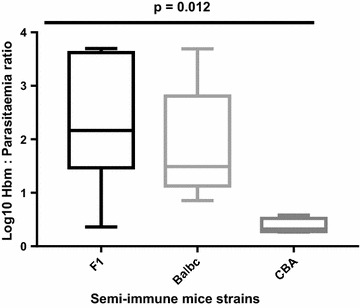
Effect of parasitaemia on minimum Hb within the semi-immune mice strains. Minimum Hb is the Hb at which low Hb is obtained during the profile of malaria anaemia in the semi-immune mice strains (Fig. 3). Parasite level at which the minimum Hb was attained was noted. The impact of parasite density on respective minimum Hb (Hbm) was compared hence the Hbm/parasitaemia. These are values of one experiment. Similar observations were made in a repeat experiment. F1 (11), Balb/c (5) and CBA (4). Middle lines within the box are median values while whiskers refers to the minimum and maximum values. One way ANOVA was used to analyse data with Tukey post-test, where p values for F1 vs. Balb/c (p = 0.562), F1 vs. CBA (p = 0.009) and Balb/c vs. CBA (p = 0.114)

Haemoglobin levels fell to 48.6, 54.0 and 62.7% of normal levels in F1, Balb/c and CBA, respectively, on days with minimum Hb (Hbm), Table 1. These Hb reductions were not significantly different within the three mice strains (p = 0.088). However, the mean parasitaemia at which this Hb reductions occurred within F1, Balb/c (< 4.0%) and CBA (27.5%) were significantly different, p = 0.012. Moreover, this minimum percentage Hb drop occurred at about the same period for each semi-immune mouse strain (p = 0.52, Table 1). Reticulocyte count was estimated in the semi-immune mice to assess extent of erythropoietic response. Similar reticulocyte count (p = 0.427) was recorded in the three semi-immune mice strains. The extent of reticulocyte production to Hb loss in the three semi-immune mice strains in this study was shown to be similar, p = 0.240. During the repeated cycles of infection and treatment, it was observed that relatively higher RBC destruction occurred at later cycles (semi-immune status of mice), but at low parasitaemia in comparison with the first cycle of infection, implying iRBC are not solely responsible in addition to inadequate reticulocyte response. With this observation, the extent to which destruction occurred in the semi-immune mice strains at the final cycle and the contribution of parasitaemia was evaluated. Figure 5 revealed that, the mean percentage Hb loss per parasitaemia was significantly different statistically in the semi-immune mice strains (p = 0.012).

**Table 1:** Hb reduction, peak reticulocyte count, and peak parasitaemia levels in the semi-immune mice strain on day minimum Hb was observed

Parameters	F1	Balb/c	CBA	p value^#^
N	11	5	4	–
Mean Hb reduction (SD)	48.6 (11.2)	54.0 (5.0)	62.7 (9.6)	0.088
Mean parasitaemia, % (SD)	3.5^a^ (9.3)	2.6 (3.0)	27.5 (7.7)	*0.012*
Mean reticulocyte level, % (SD)	19.9 (8.5)	35.8 (17.8)	18.8 (15.1)	0.427
Mean of reticulocyte level/Hb reduction (SD)	0.41 (0.2)	0.64 (0.3)	0.28 (0.3)	0.240
Mean period, day (SD) at which Hb_m_ was observed	13.4 (3.2)	15.2 (2.0)	13.5 (2.2)	0.520

These are values of one experiment. Hb reduction is the difference between %Hb on day prior to first cycle infection and treatment, and day on which Hb_m_ (minimum Hb). Hb_m_ is the minimum Hb at which blood was harvested for serum. Similar observations were made in a repeat experiment

*SD* standard deviation

^#^One-way ANOVA with Tukey’s post-test

^a^Significantly lower than CBA

### Antibody levels, T cell responses and cytokines levels in the semi-immune mice strains

**Fig. 6:**
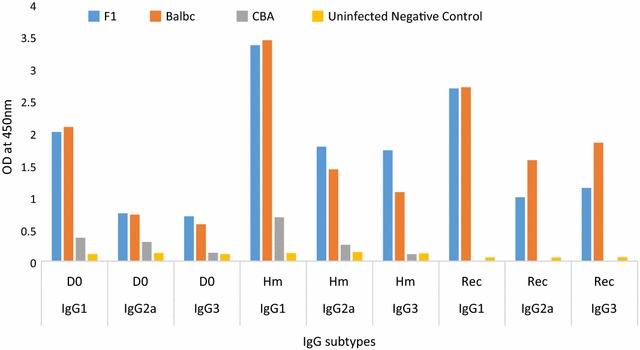
IgG subtypes in the semi-immune mice strains. Serum was harvested on D0, Hm (day 16 for F1, Balb/c and day 12 for CBA) and Rec (day 28 for F1 and Balb/c). IgG subtypes (IgG1, IgG2a and IgG3) were measured (in triplicates) on the pooled sera for each semi-immune mice strains on the days serum was harvested. Values presented are the mean of the triplicates. D0 was the start of the last challenge, while Hm was when minimum Hb is attained, Rec is when parasitaemia was zero by microscopy. Mice number: F1 (5); Balb/c (5), CBA (5) and Uninfected negative control (3). *D0* day zero, *Hm* day minimum *Hb* observed, *Rec* day parasite not detected by microscopy (parasitaemia is zero by microscopy)

To further explore the role of other immune mechanism and at what point the immune system becomes more aggressive, in relation to the recovery by F1 and Balb/c and death in the CBA, the kinetics of IgG subtypes and T cell populations were assessed. IgG subtypes were generally high in the semi-immune mice strains when compared to the uninfected negative control suggesting the antibody production was initiated by the parasite infection (Fig. 6). Furthermore, IgG subtypes were highest at the point when minimum Hb (Hm) was observed in the three mice strains. In all the three points that antibody type was measured, IgG1 was highest in F1 and Balb/c than CBA. Finally, IgG subtypes were more than two times higher in the F1, Balb/c than the CBA semi-immune mice strains, at D0 and Hm (Additional file 1).

**Fig. 7:**
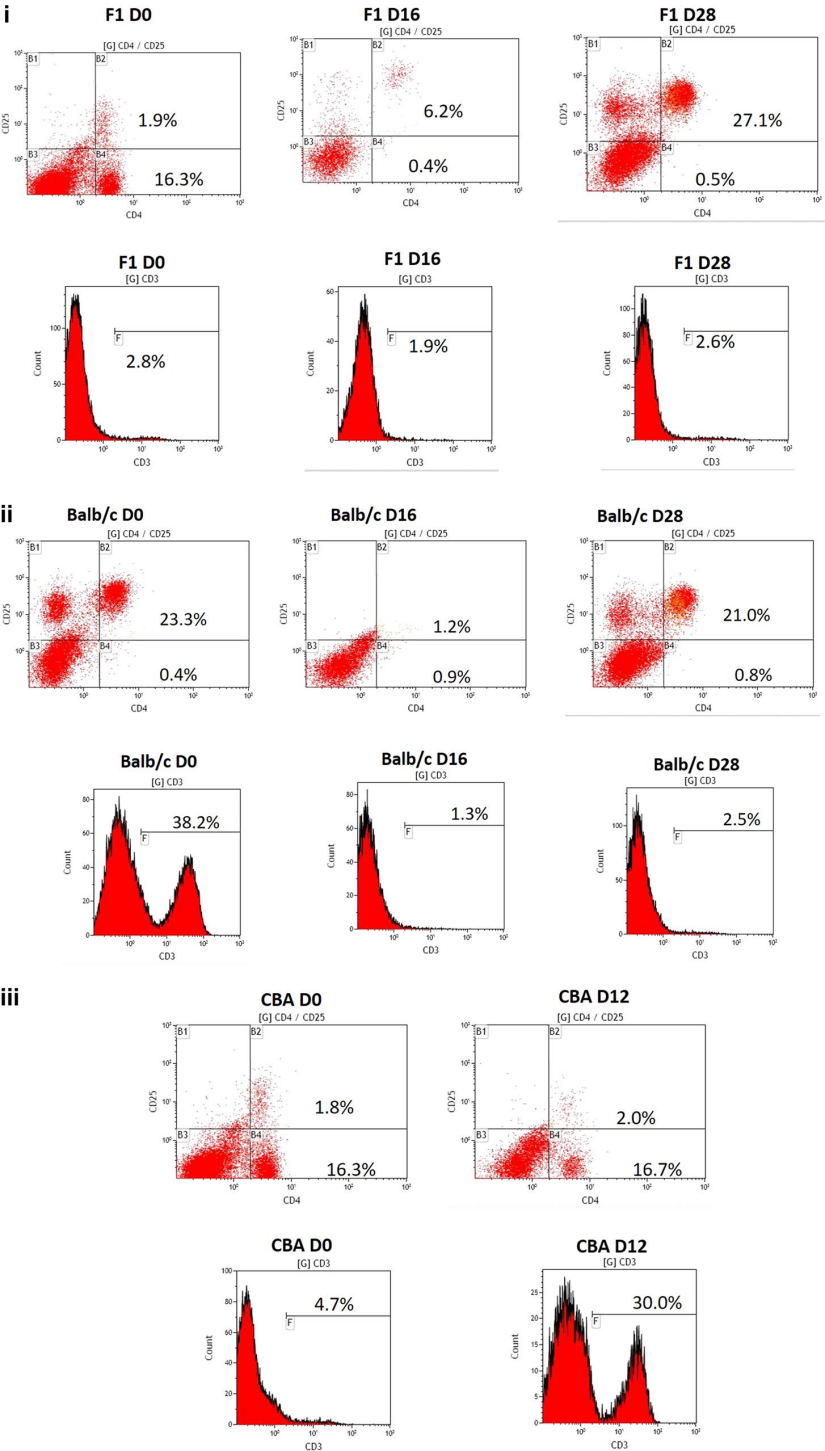
T cell population in the semi-immune mice strains. Mice were sacrificed and spleen removed on days 0, minimum Hb (Hm) [day 12 for CBA and day 16 for F1 and Balb/c], and when mice recovered (i.e. when parasitaemia is zero by microscopy), day 28 for F1 and Balb/c. Most CBA died by this time. T cell population were analysed using the flow cytometry. FACs analysis was done on pooled cells from the mice [F1 (5), Balb/c (5) and CBA (5), Negative control (3)] sacrificed in each experiment. The negative values (i.e. negative controls) were subtracted and values are presented for the positives. *D0* day zero, *Hm* day minimum *Hb* observed, *Rec* day parasite not detected by microscopy, *T cells* pooled cells from the semi-immune mice and done in triplicates. Values presented are the mean of the triplicates. A representative result of the FACs analysis for **i** F1; **ii** Balb/c; **iii** CBA

**Fig. 8:**
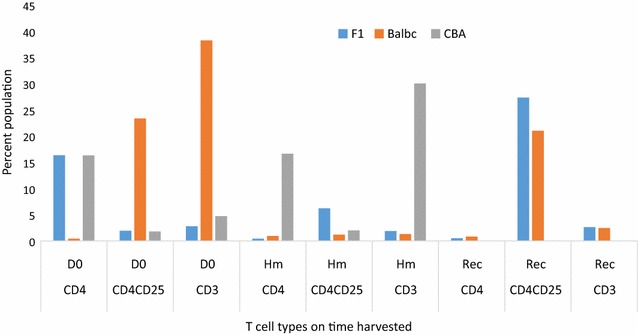
T cell population in the semi-immune mice strains. Mice were sacrificed and spleen removed on days 0, minimum Hb (Hm) [day 12 for CBA and day 16 for F1 and Balb/c], and when mice recovered (i.e. when parasitaemia is zero by microscopy), day 28 for F1 and Balb/c. Most CBA died by this time. T cell population were analysed using the flow cytometry. FACs analysis was done on pooled cells from the mice [F1 (5), Balb/c (5) and CBA (5), Negative control (3)] sacrificed in each experiment. The negative values (i.e. negative controls) were subtracted and values are presented for the positives. *D0* day zero, *Hm* day minimum *Hb* observed, *Rec* day parasite not detected by microscopy, *T cells* pooled cells from the semi-immune mice and done in triplicates. Values presented are the mean of the triplicates. T cell (CD3, CD4 and, CD4^+^25^+^) levels in the F1, Balb/c and CBA semi-immune mice strains

Kinetics of T cell populations in the three semi-immune mice strains revealed that while CD4 T cells were very low in Balb/c semi-immune mice on day zero, CD4^+^CD25^+^ and CD3 were much higher on day zero (D0) (Figs. 7, 8). These T cell types were very low at Hm (minimum Hb), with only CD4^+^CD25^+^ increasing sharply again at recovery in the Balb/c semi-immune mice (Additional file 1). F1 on other hand had very high CD4 T cell, with very low CD4^+^CD25^+^ and CD3 levels recorded on D0. Levels of CD4 T cell was drastically reduced with an increase in CD4^+^CD25^+^ and CD3 at minimum Hb (Hm) for F1. Interestingly, there was a sharp increase in the CD4^+^CD25^+^ with low CD4 and CD3 T cell types in the F1 semi-immune mice at recovery (Rec), Figs. 7, 8. Since CBA semi-immune mice did not recover and died due to high parasitaemia, T cell type assessment was done at two-time points, Figs. 7iii and 8. While T cells expressing CD4 in CBA was high and similar at D0 and Hm, CD4^+^CD25^+^ was low on both days (D0 and Hm in the CBA semi-immune mice). CD3 T cells was, however, much higher at Hm. Another interesting finding is that T cells expressing CD4 and CD3 were highest in the CBA semi-immune mice strains than the F1 and Balb/c semi-immune mice strains at Hm.

As IgG subtypes were elevated at Hm with relatively low T cell population observed at the Hm (F1 and Balb/c in particular), the role of anti- and pro-inflammatory cytokines levels were evaluated at the Hm (Table 2). Hm was the point where low Hb was observed in the last cycle of infection without treatment (Fig. 1). In F1 and Balb/c semi-immune mice, this was the time the mice start clearing the parasite by themselves (without treatment, Fig. 1) suggestive of protection, thus IL-17 was measured together with the other anti- and pro-inflammatory cytokines that are thought to be involved in suppressing erythropoiesis, and others contributing to anaemia in children with falciparum malaria [33, 34]. It was observed that significantly high levels of IL-4, IL-12, IL-17 and IFN-γ were recorded in the F1 and Balb/c mice strains, p < 0.05 (Table 2). The other cytokines and the TNF: IL-10 ratio was comparable in the three semi-immune mice strains studied in this current paper (p > 0.05), Table 2.

**Table 2:** Cytokine levels in the semi-immune mice of Balb/c, F1 and CBA

Cytokine (pg/mL)	Mouse strains (number)				p value*
	F1 (n = 5)	Balb/c (n = 5)	CBA (n = 5)	Uninfected control (3)	
IL-1α (SD)	1.0 (0.2)	1.4 (0.6)	1.2 (0.1)	0.9 (0.1)	0.244
IL-4 (SD)	0.8 (0.1)	0.8 (0.1)	0.6 (0.1)	0.5 (0.1)	*0.017*
IL-10 (SD)	1.3 (0.1)	1.4 (0.4)	1.6 (0.2)	1.3 (0.2)	0.125
IL-12 (SD)	3.2 (0.2)	3.4 (0.2)	2.9 (0.5)	2.6 (0.2)	*0.018*
IL-17 (SD)	3.1 (0.4)	3.3 (0.7)	0.8^a^ (0.04)	0.7^a^ (0.05)	*<* *0.0001*
IFN-γ (SD)	0.8 (0.1)	1.3^b^ (0.3)	0.8 (0.1)	0.6 (0.04)	*0.002*
TNF-α (SD)	1.5^c^ (0.1)	1.6 (0.2)	1.8 (0.2)	1.6 (0.04)	0.066
TNF-α: IL-10 (SD)	0.2 (0.2)	0.2 (0.4)	0.1 (0.1)	0.3 (0.2)	0.874

Blood was harvested when mice were sacrificed at minimum Hb. Values are results of one experiment. Similar results were obtained in a repeat experiment

* p values done by ANOVA was performed on the log_10_ transformed data. Analysis was done by ANOVA with Tukey’s post test. Values represented are mean of the log_10_ transformed data with standard deviation (SD)

^a^Significantly lower than F1 and Balb/c

^b^Significantly higher than the other strain and the negative control

^c^Significantly lower than CBA

## Discussion

Studies have shown that malaria anaemia at low parasitaemia occurs in malaria endemic areas as individuals have become immune or semi-immune [23, 33]. Mice studies have modeled recovery/survival from malaria infection, and factors associated with that include destruction of uninfected RBC (uRBC) [23, 32] and elevated levels of anti- erythropoietin antibodies [24] among others; and in humans elevated levels of some pro-inflammatory cytokines (e.g., IFN-γ and TNF) have been observed to be protective [10, 12]. High levels of IL-17 cytokines have been observed in SMA cases [26, 27], but no studies of IL-17 association with recovery from plasmodium infection at low Hb in the semi-immune has been explored even though IL-17 has been noted to be protective in some infections [17]. Results from this study indicates that elevated levels of IL-17 together with IL-4, IL-12 and IFN-γ are observed in semi-immune mice that recovered from malaria infections via Hb loss, and certain genes may be involved.

Crossing of Balb/c and CBA to get the F1 generation was to assess the role of a possible candidate gene that might be responsible in eliciting destruction of high amount of uRBC via some immune mediated mechanism resulting in malaria anaemia. The survival (Fig. 2), extent of Hb loss (Table 1) and effect of parasitaemia on Hb loss (Fig. 5) were similar in the Balb/c and F1 semi-immune mice. These observations mirror a previous report [23] by the authors, and further implicating an immune mediated mechanism (elevated levels of anti-erythropoietin antibodies) resulting in chronic low Hb [24], suggesting two ways in lowering the Hb, which are RBC destruction (both infected and uninfected) and suppression of erythropoiesis. These findings strengthen the consistency and reproducibility in earlier studies by the authors and the hypothesis generated that low Hb observed in malaria infections is partly due to high destruction of uRBC, which is likely controlled by a gene(s), and was passed on from Balb/c to the F1 progeny. The chronic low Hb creates an environment of limited iron bio-availability for the *Plasmodium* parasite to proliferate, hence the recovery at low Hb (Hm).

High parasitaemia in CBA resulting in their death might be explained by the lack of efficient control of plasmodium parasites growth. This is stemmed from the observation of relatively low IgG subtypes in CBA semi-immune mice. IgG subtypes were, however, higher in the Balb/c and F1 semi-immune mice strains. These IgG subtypes are important in enhancing the efficient control of Plasmodium parasitaemia either through the immune mechanism of RBC destruction and/or direct destruction of the infected RBCs in addition to the suppression of erythropoiesis. Furthermore, studies have shown that Th1 cytokine response made up TNF, IFN-γ, IL-1 and IL-6 are required for the activation of immune cells against malaria infection [34, 35]. It is postulated that CBA attempts to fight the high parasitaemia via CD3 T cells in association with CD4 T cells, a helper T cell. The higher CD3 T cells recorded for CBA at low Hb (Hm) suggests an auto-immune involvement. Anti-CD3 antibodies have been shown to ameliorate the symptoms of auto-immune disorders [36]. This suggests that CD3 levels are important in auto-immune activities. Thus, much higher levels of CD3 was expected in Balb/c and F1. However, the converse is the case. The extent of auto-immune involvement in CBA to lower the parasitaemia needs to be explored. It is postulated that the parasite growth in the CBA initiates a sustained high level of CD4 T cells from Day 0 till minimum Hb is observed (Hm, Figs. 7, 8). This is not surprising as T helper cells (identified by CD4 marker), will obviously be involved in such immune activity. Balb/c and F1 on the other hand use a concerted effort of higher and sustained level of IgG levels together with regulatory cells at D0. The T regulatory cells (CD4^+^CD25^+^), then get lowered at Hm, where IgG subtypes gets higher to keep the parasite at bay (low or zero parasitaemia by microscopy). After the Hm (i.e. at recovery, Rec), T regulatory cells increases again, which is necessary to limit and control the hyper immune activity. With the high IL-17 levels in Balb/c and F1 at the minimum Hb point, it is possible that IL-17 is involved in mobilizing IgG subtypes to limit rapid Plasmodium proliferation in the Balb/c and F1. Furthermore, IL-17 may act to generate cellular foci to contain chronic infection as modelled in the semi-immune mice used in this and other studies [23, 24, 32] and also observed in the malaria endemic areas. Interestingly, two recent studies reported a significantly lower IL-17 in SMA cases compared with mild malaria anaemia (MMA) [26, 27]. This is in contrast with the studies of higher IL-17 levels in the anaemic semi-immune mice. The current study mirrors a chronic situation at recovery, and this might contribute to the differences observed in this current study and the other two [26, 27]. Nevertheless, the results presented here can be relevant for malaria infection in general, which has been explored in other studies [13–15].

Meanwhile some studies report of high plasma levels of IL-17 with increase in mortality [15], and low levels of IL-10 and TGF-β due to *Plasmodium* infection [37]. However, this current study reports of significantly higher IL-17 as well as high IL-12, IL-4 and IFN-γ in the Balb/c and F1 semi-immune mice with zero/low parasitaemia (by microscopy) during Rec. The high IL-17 level and high survival reported in this current study is at variance with what is reported by [15]. It is not clear if the model of semi-immune status described in the current study might be a contributory factor. Furthermore, it is suspected that high IL-17 levels alone is not enough to contribute in the high survival, but in association with high levels of IL-12, IL-4 and IFN-γ. IL-10 in the current study was similar in the three mice strains used in the study. It is not clear what might have resulted in the differences between the results presented here and that of [37]. It is tempting to speculate that pregnancy might have affected the extent of cytokine production, as pregnant mice were used in that study by [37]. Significantly high levels of IL-4, IL-12 and IFN-γ together with IL-17 in the anaemic mice (Balb/c and F1) reported in the current study, suggests suppression of erythropoiesis and/or destruction of uRBC leading to the low Hb consistently observed. Similar observation was made in an earlier report concerning high levels of IFN-γ association with low Hb [24, 38]. Further studies are needed to clarify the mechanisms involved in this association. Similar levels of IL-10, TNF among the semi-immune anaemic mice (Balb/c and F1) in the current study was also observed in an earlier study [24]. This observation was surprising since TNF and IL-10 are thought to contribute to the degree of anaemia in children with falciparum malaria [39, 40]. The results presented in this report also suggests that IL-1α, a protective cytokine, is not associated with protection in Balb/c and F1, even though an association of IL-1α with protection has been reported previously [41]. Finally, since more of IL-12, IFN-γ (Th1 cytokines) were significantly higher in the semi-immune anaemic mice (Balb/c and F1), it is hypothesized that at low Hb (Hbm) mechanism of recovery or protection from the Plasmodium infection is shifted towards Th1 response.

From the data presented here it can concluded that certain genes are contributing to the extent of survival (protection) against Plasmodium infection through low Hb. And the mechanism employed in this protection against the Plasmodium infection may be more of a Th1 response at low Hb (Hbm) where F1 and Balb/c start clearing the parasites without treatment (Rec). Despite the strengths in this study, there were some limitations. These were the fact that pooled sera were used in analysing the IgG subtypes as well as T cell types analysis was done on pooled splenocytes. Also, the fact that Th17 cells were not assessed. Finally, even though the small number of CBA mice used might be a limitation, the results can still be valuable considering the fact that similar results were obtained in repeat experiments in this study. These can be areas for further studies. Further studies looking into the role of IL-17 in Balb/c will be informative in understanding the promising protective role of IL-17. In addition, further studies of F2 between the F1 and Balb/c will also be informative in evaluating if these genes are segregated or further apart.

## Additional file

**Additional file 1.**
